# Indications of medical plants: what do medical students in Germany know? A cross-sectional study

**DOI:** 10.1007/s00432-022-03921-6

**Published:** 2022-01-29

**Authors:** Sören Klaus Büntzel, Maria-Louisa Ritschel, Rebecca Wurm-Kuczera, Judith Büntzel

**Affiliations:** 1 University Medical Center Magdeburg, Otto-Von-Guericke University, Magdeburg, Germany; 2 Department of Hematology and Medical Oncology, University Medical Center Goettingen, Georg-August University, Göttingen, Germany; 3 Working Group “Prevention and Integrative Oncology”, German Cancer Society, Berlin, Germany

**Keywords:** Phytotherapy, Medical education, Complementary and alternative medicine, Medical plants

## Abstract

**Background:**

Patients use phytotherapy in addition to cancer therapy. Aiming to equip students with the knowledge necessary for a holistic treatment approach, the (German) national competence-based catalogue of educational objectives in medicine advises to include phytotherapy as part of the curriculum. Here, we evaluate if medical students know the official indication of herbal products as stated by the German Federal Institute for Drugs and Medical Devices (BfArM) for treating oral and abdominal discomfort.

**Methods:**

In an online survey, students were asked to write down indications they associated with 25 plants used for treating either oral or abdominal discomfort. Students’ indications were then sorted according to symptom complexes (oral discomfort, abdominal discomfort, skin care, others) and compared with the official indications stated by the BfArM.

**Results:**

Of 168 participating students, 113 wrote down indications for 22 of the 25 plants surveyed. 70.80% of the students knew the correct indication for Matricaria recutita, 41.59% for Salvia officinalis, 37.17% for Foeniculum vulgare Mill. and 36.28% for Mentha piperita. Those were the highest rates of correct answers. Looking at symptom complexes (e.g., oral/ abdominal discomfort) instead of single symptoms, ≥ 10% of the students stated an indication that fell into the same symptom complex as the BfArM’s indication for 10 out of 25 plants surveyed. The most well-known plants and indications were Matricaria recutita, Salvia officinalis and Mentha x piperita.

**Conclusion:**

Only a small minority of participating students know the official indications for specific phytotherapeutic drugs. Our study demonstrates that phytotherapy needs to be incorporated into the medical teaching curriculum.

## Introduction

While 40–90% of all cancer patients use complementary or alternative medicine (CAM) in addition to conventional oncological treatment in the hope of alleviating side effects of cancer therapy (Micke et al. 2009; Huebner et al. 2014; Wortmann et al. 2016), more than two-thirds of patients do not inform their physician about their use of CAM (Micke et al. 2009). Furthermore, instead of asking a health care practitioner for information regarding CAM, most patients use friends, family or media outlets as sources of information (Molassiotis et al. 2005; Huebner et al. 2014). Even if they actively seek out professionals, questions are usually directed toward pharmacists and general practitioners but not the treating oncologist (Micke et al. 2009). Unfortunately, both pharmacists and physicians do not feel well equipped to sufficiently counsel their patients due to a lack of knowledge and evidence-based, professional resources (Ventola 2010). We know that herbs are among the most used CAM treatments (Molassiotis et al. 2005; Huebner et al. 2014). Here, we observe a gap between the daily life of our patients that are using phytotherapy and the skill of medical staff to carefully advice patients on advantages but also pit-falls of using medical plants. If we wish to equip physicians with this particular knowledge and tools to advice patients, we require a broad, standardized and evidence-checked approach for teaching about phytotherapy.

One logical choice would be to integrate phytotherapy into the curriculum of medical schools. Medical students seem to be motivated to learn about medical plants and CAM. A small survey among medical students showed that students think that physicians should be able to answer patients’ questions about phytotherapy. A recent study showed that students are interested to study CAM as a part of their curriculum and to improve their knowledge on CAM (Flaherty et al. 2015). Students themselves use medical plants while at the same time recognizing existing misinformation about phytotherapy (Enwere 2009). Like the pharmacists and physicians cited above (Ventola 2010) medical students encounter patients using herbals, but are unable to advise patients on the pit-falls and indications of phytotherapy (Xu and Levine 2008).

Even if medical staff and medical students are not secure how to talk and advice about CAM and phytotherapy (Enwere 2009; Ventola 2010), we should not ignore knowledge on medical plants. Phytotherapy has a long-standing tradition in Europe, exemplified by accepting herbal knowledge in Europe as the heritage of mankind—recently, the wisdom of Pinzgau herb woman was added to the Austrian World heritage list (Buchart 2010). The German Federal Institute for Drugs and Medical Devices (BfArM) monitors usage and provides indications for using herbal medicinal products [*BfArM—Complementary and Alternative Medicines (CAM) and Traditional Medicinal Products (TMP)*, n.d.]. Herbal products also fall under the European Medicine Agency (EMA) Guideline on Quality of Herbal Medicinal products (EMA/CPMP/QWP/2819/00). The guideline also includes traditional medicinal plants, if the herb’s use is well established or if the herb is in use for more than 30 years in Europe (Fürst and Zündorf 2015). In Germany, traditional herbal remedies fall under the German Medicines Act. The BfArM monitors usage and provides indications for using herbal medicinal products. Indications are mapped out by experts of the “Commission E” [*BfArM—Complementary and Alternative Medicines (CAM) and Traditional Medicinal Products (TMP)*, n.d.]. Summing up, the indications for using medical plants in Germany are mandated by the BfArM similarly to the indications of drugs. These official indications offer a common base of knowledge and application.

If these official indications of medical plants are known, medical staff should be able to correctly advice their patients. However, we still not know of the current status of knowledge. As mentioned above, integrating phytotherapy into the curriculum of medical schools would offer a broad, standardized and evidence-checked approach to teach about phytotherapy. Therefore, we assessed the knowledge of medical students on medical plants in a first step. In a second question we asked whether there are already structures integrating CAM and phytotherapy into the curriculum of medical schools in Germany.

## Methods

### Survey and participants

Two investigators (SKB and M-LR) assessed whether naturopathy or phytotherapy are taught in German medical schools by screening curricula, timetables and study guides for electives of 38 medical schools. This step was undertaken under the following premise: (1) if phytotherapy is taught and students do not know of the medical plants official indications, the curricula of medical schools should be improved and (2) if phytotherapy is not taught at all, this would explain, if the knowledge of students on the topic of medical plants was lacking.

The second focus of this study was to appraise the knowledge of medical students on the official indications of medical plants used in Germany. We chose “oral discomfort” and “abdominal discomfort” as exemplary symptoms. Both symptom complexes were chosen due to their common occurrence in patients undergoing chemotherapy.

We used our previously published method to select plants used in this survey (Buentzel et al. 2020). Books used to generate “hit-lists” of plants for treating oral and abdominal discomfort are listed in Table 1. The plants most often mentioned by literature were used to generate a list of medical plants used for treating either oral or abdominal discomfort. Plants surveyed are listed in Table 2. This list of plants was then used for an anonymous online survey assessing knowledge on traditional medical plants. The questionnaire comprised the German designation and a picture each plant surveyed.

**Table 1:** German books screened for plants used for treating oral and abdominal discomfort

Books on phytotherapy screened for plants treating bloating
Achmüller A (2018) Verdauung und Entschlackung, 1st ed. Edition Raetia
Anonymous (2013) Heilpflanzen: Erkennen, sammeln und anwenden. Neuer Kaiser
Hoffmann P (2019) Lexikon der Arzneipflanzen: Wegweiser zur Selbstbehandlung. Nikol, Hamburg
Pahlow AM (2004) Das große Buch der HEILPFLANZEN. Weltbild
Ploss DO (2007) Klostermedizin—Die 50 besten Tipps: Klosterheilkunde neu entdeckt 50 Alltagsbeschwerden und Krankheiten von A bis Z. Knaur Kreativ
Seitz P, Engelberth J (1996) Heil- und Gewürzpflanzen aus dem eigenen Garten. AID. Bonn
Siewert AM (2019) Gesund älter werden mit den besten Heilpflanzen: Tees, Tinkturen, Präparate & Anwendungen für mehr Lebensenergie, 2nd ed. GRÄFE UND UNZER Verlag GmbH, München
Stange R, Kraft K (2009) Lehrbuch Naturheilverfahren, 1st ed. Hippokrates, Stuttgart
Steigerwald P-A (2015) Phytotherapie pocket, 3rd ed. Börm Bruckmeier, Grünwald
Throll A, Tomsky J (2014) Das Kräuterwissen der Apotheker: Heilpflanzen-Rezepte für meine Hausapotheke, 1st ed. Franckh Kosmos Verlag, Stuttgart
Wenigmann M (2017) Phytotherapie: Arzneidrogen—Phytopharmaka—Anwendung, 1st ed. Urban & Fischer Verlag/Elsevier GmbH, München
Books on phytotherapy screened for plants treating constipation, diarrhea and nausea
Achmüller A (2018) Verdauung und Entschlackung, 1st ed. Edition Raetia
Hoffmann P (2019) Lexikon der Arzneipflanzen: Wegweiser zur Selbstbehandlung. Nikol, Hamburg
Hensel W (2020) Welche Heilpflanze ist das?, 4th ed. Franckh Kosmos Verlag, Stuttgart
Madejsky M (2019) Praxishandbuch Frauenkräuter: Mit vielen Rezepten und praktischen Heilpflanzen-Anwendungen. Frauenheilkunde aus der Natur, 1st ed. AT Verlag
Mayer JG, Uehleke B, Saum PK (2013) Das große Buch der Klosterheilkunde, 1st ed. ZS Verlag Zabert Sandmann GmbH, München
Ploss DO (2007) Klostermedizin—Die 50 besten Tipps: Klosterheilkunde neu entdeckt 50 Alltagsbeschwerden und Krankheiten von A bis Z. Knaur Kreativ
Schaffner W (1996) Pflanzenführer, Heilpflanzen Kompendium, Vorkommen, Merkmale, Inhaltsstoffe, Anwendung. Naturbuchverlag, Augsburg
Schönfelder P, Schönfelder I (2019) Der Kosmos Heilpflanzenführer: Über 600 Heil- und Giftpflanzen Europas, 4th ed. Franckh Kosmos Verlag, Stuttgart
Stumpf U (2021) Unsere Heilkräuter: bestimmen und anwenden, 3rd ed. Kosmos, Stuttgart
Wenigmann M (2017) Phytotherapie: Arzneidrogen—Phytopharmaka—Anwendung, 1st ed. Urban & Fischer Verlag/Elsevier GmbH, München
Books on phytotherapy screened for plants treating oral discomfort
Achmüller, A (2012) Teufelskraut, Bauchwehblüml, Wurmtod: das Kräuterwissen Südtirols: Mythologie, Volksmedizin und wissenschaftliche Erkenntnisse, Edition Raetia, Bozen
Hensel W (2020) Welche Heilpflanze ist das?, 4th ed. Franckh Kosmos Verlag, Stuttgart
Landespflege, Bayerischer Landesverband f Gartenbau, Hohenberger E, Votteler W (2017) Gewürzkräuter und Heilpflanzen. 7th ed, Obst- und Gartenbauverlag des Bayerischen Landesverbandes für Gartenbau und Landespflege e.V, München
Mayer JG, Uehleke B, Saum PK (2013) Das große Buch der Klosterheilkunde, 1st ed. ZS Verlag Zabert Sandmann GmbH, München
Niederegger, O, Mayr C (2005) Hausbuch der Südtiroler Heilkraeuter Gesundheit aus der Natur, Athesia, Bozen
Pahlow AM (2004) Das große Buch der HEILPFLANZEN. Weltbild
Prentner, A (2017) Heilpflanzen der Traditionellen Europäischen Medizin: Wirkung und Anwendung nach häufigen Indikationen, Springer-Verlag, Berlin
Rätsch, C (2014) Heilpflanzen der Antike: Mythologie, Heilkunst und Anwendung, AT Verlag, Aarau
Stange R, Kraft K (2009) Lehrbuch Naturheilverfahren, 1st ed. Hippokrates, Stuttgart
Steigerwald P-A (2015) Phytotherapie pocket, 3rd ed. Börm Bruckmeier, Grünwald

**Table 2:** Plants surveyed

Oral discomfort	Abdominal discomfort
*Althaea officinalis* L.	*Foeniculum vulgare* Mill.
*Angelica archangelica* L.	*Linum* L.
*Artemisia absinthium* L.	*Mentha* x *piperita* L.
*Calendula officinalis* L.	*Pimpinella anisum* L.
*Centaurium* Hill.	*Plantago ovata* Forssk.
*Cetraria islandica* Arch.	*Quercus robur* L.
*Cichorium intybus* L.	*Rheum* L.
*Cuminum cyminum* L.	*Senna* L.
*Gentiana lutea* L.	*Vaccinium myrtillus* L.
*Malva sylvestris* et *neglecta*	
*Matricaria recutita* L.	
*Plantago lanceolata* L.	
*Potentilla erecta* Raeusch	
*Salvia officinalis* L.	
*Taraxacum* sect. *Ruderalia Kirschner*	
*Zingiber officinale* Roscoe	

The project was approved by the local ethic committee of the medical faculty in Jena (approval numbers: 2020-1866-Bef, 2020-1881-Bef). If medical students recognized a medical plant, they were asked to write down the indication they associated with this medical plant. Metric data were obtained on whether students were enrolled in pre-clinical or clinical courses and whether medical plants were part of their faculties’ curriculum. The online tool used for generating and hosting the survey was https://soscisurvey.de. The survey recruited between March and April 2020. Microsoft Excel 2010 was used for calculations and data management.

### How to appraise the knowledge of medical students on traditional German herbal medicine?

The official indications of medical plants are published by the BfArM—either as monographs or as official overview tables. Indications are clearly marked as such within the monographs and overview tables. We retrieved these indications from the monographs and overview tables. Indications were cross-checked with a pharmaceutical book on German phytoherapy (Steigerwald P-A (2015) Phytotherapie pocket, 3rd ed. Börm Bruckmeier, Grünwald).

Students were recruited via social media. The participating students were presented the German designation and a picture of each plant surveyed. They were asked to write down for which indication they (would) use any of the plants presented in their daily life. Students were also given an example for filling out the questionnaire (e.g., Foeniculum vulgare—abdominal discomfort).

We then compared students’ indications with the official indications published by BfArM.

Both, students’ and the BfArM’s indications were sorted for data analysis. A correct answer was given, if the BfArM’s indication matched at least one indication of the students’ answer(s). If a student’s indication was not the same as the official indication of the BfArM, investigators reviewed whether both indications fell into the same symptom complex. Symptom complexes were defined as followed:

1. Oral discomfort: oral mucositis, loss of appetite, xerostomia, dysgeusia.
2. Abdominal discomfort: dyspepsia, bloating, constipation, diarrhea.

From the information retrieved, we calculated the rates of knowledge of “official indications” and “symptom complexes”. Rate of knowledge of official indications was calculated dividing the number of students that knew at least one correct indication per plant by the number of all students participating. Rate of knowledge of symptom complexes was calculated similarly.

Summarizing, we analyzed data in a two-step process looking for (1) total congruency between indications (students stating the same indication as the BfArM) and (2), in the case of no congruency, if at least students’ and the BfArM’s indications fell into the same symptom complex.

## Results

### Characteristics of participating students

In spring 2020, we launched an online survey questioning German medical students about their knowledge on traditional medical herbs used to treating abdominal discomfort and symptoms associated with oral mucositis. Of 168 students participating in the survey only 113 wrote down various indications. 65 (38.69%) students were enrolled in pre-clinical and 78 (46.43%) in clinical courses and 11 (6.55%) students were already taking their practical year. 151 (89.89%) were medical students, one was a dental student, ten (5.95%) were “other”, and six participants did not indicate their occupation. 55 (32.74%) students stated that phytotherapy was a part of their faculty’s main curriculum and 39 students (23.21%) noted that knowledge on medical herbs was covered by electives. However, over a third of the students stated that phytotherapy was not taught at their faculty.

### Knowledge on traditional usage is present, but not in line with official indications

25 plants were included in our survey. Students only wrote down indications they associated with plants they knew and used in daily life, meaning that students only considered a small selection of plants while answering. However, regarding all answers of the 113 students writing down indications, at least one correct BfArM indication per plant was stated for 22/25 plants in our sample. Next, we focused on single plants. The highest rate of stating the correct indication for single drug use was accounted for *Matricaria recutita* L. (known to 70.80% of the students), *Foeniculum vulgare* Mill. (known to 37.17% of the students), *Salvia officinalis* L. (known to 41.59% of the students) and *Mentha* × *piperita* L. (known to 36.28% of the students). For herbal remedies used in a standardized mixture, the most known plants were for *Foeniculum vulgare* Mill. (known to 37.17% of the students), *Matricaria recutita* L. (known to 28.32% of the students), *Pimpinella anisum* L. (known to 16.81% of the students) and *Plantago ovata* Forssk. (known to 12.39% of the students). Only a small minority of students was aware about the official indications (Table 3).

**Table 3:** Knowledge of students (*N* = 113) on indications of medical plants used for treating oral and abdominal discomfort

Plant	Single plant or mixture	Number of official indications*	Degree of knowledge (official indications*)	Degree of knowledge (symptom complex)
*Althaea officinalis* L.	Single plant	4	0.88%	2.65%
	Mixture	3	2.65%	2.65%
*Angelica archangelica* L.	Single plant	5	0.00%	0.00%
	Mixture	5	0.00%	0.00%
*Artemisia absinthium* L.	Single plant	4	3.54%	3.54%
	Mixture	6	3.54%	3.54%
*Calendula officinalis* L.	Single plant	3	7.08%	23.89%
*Centaurium* Hill.	Single plant	3	0.00%	0.00%
*Cetraria islandica* Arch.	Single plant	2	0.88%	26.55%
	Mixture	3	9.73%	26.55%
*Cichoricum intybus* L.	Single plant	3	0.00%	0.00%
*Carum carvi* L.	Single plant	4	8.85%	9.73%
	mixture	5	8.85%	9.73%
*Foeniculum vulgare* Mill.	Single plant	6	37.17%	39.82%
	Mixture	7	37.17%	39.82%
*Gentiana lutea* L.	Single plant	4	0.88%	0.88%
	Mixture	5	0.88%	0.88%
*Linum* L.	Single plant	3	30.09%	30.09%
*Malva sylvestris* et *neglecta*	Single plant	3	0.88%	0.88%
*Matricaria recutita* L.	Single plant	10	70.08%	77.88%
	Mixture	4	28.32%	28.32%
*Mentha* x *piperita* L.	Single plant	9	36.28%	40.71%
	Mixture	6	6.19%	7.08%
*Pimpinella anisum* L.	Single plant	4	12.93%	18.58%
	Mixture	5	16.81%	19.47%
*Plantago lanceolata* L.	Single plant	6	0.88%	13.27%
*Plantao ovata* Forssk.	Single plant	3	17.70%	24.78%
	Mixture	1	12.39%	23.01%
*Pontentilla errecta* L.	Single plant	2	0.00%	0.00%
*Quercus robur* L.	Single plant	6	0.00%	0.00%
*Rheum* L.	Single plant	1	0.00%	0.00%
*Salvia officinalis* L.	Single plant	5	41.59%	65.49%
*Senna* L.	Single plant	1	0.88%	0.88%
	Mixture	1	0.88%	0.88%
*Taraxacum* sect. *Ruderalis Kirschner*	Single plant	6	0.88%	0.88%
	Mixture	4	2.65%	2.65%
*Vaccinium myrtillus* L.	Single plant	5	0.88%	0.88%
*Zingiber officinale* Roscoe	Single plant	3	6.19%	6.19%
	Mixture	4	1.77%	6.19%

^a^Indication as officially provided by the German Federal Institute for Drugs and Medical Devices

We analyzed if indications of students and the official indications of the BfArM matched the same symptom complex. Here, ≥ 10% of the students knew an indication that fell into the same symptom complex as the BfArM’s indication for 10 out of 25 plants used as single drug. Matricaria recutita L. (known to 77.88% of the students), Salvia officinalis L. (known to 65.49% of the students) and *Mentha* × *piperita* L. (known to 40.71% of the students) were the most well-known plants. For plants used in fixed mixtures with other herbs, ≥ 10% of the students stated an indication for the same symptom complex as the BfArM’s indication for 5 out of 14 remedies. Here, *Foeniculum vulgare* Mill. (known to 39.82% of the students), *Matricaria recutita* L. (known to 28.32% of the students) and *Cetraria islandica* L. (known to 26.55% of the students) were the most well-known plants. For an overview of all plants, refer to Table 3.

### Naturopathy in German medical education

Naturopathy is a part of the national competence-based catalogue of medical educational objectives. The learning objective on naturopathy does also comprise to a part phytotherapy (Medizinischer Fakultätentag der Bundesrepublik Deutschland e. V. 2015). Naturopathy is taught in 25 of the 38 (65.79%) of the German medical schools, and is part of the cross-sectional subject Q12 (“rehabilitation, physical medicine, naturopathy”) at 21 of these 25 German faculties (ÄApprO 2002*—Approbationsordnung Für Ärzte*). Eleven medical schools offer electives covering naturopathy. Six of 38 faculties offer a model medical education program, following a modified curriculum. Here, it was not possible to appraise if these faculties offer an equivalent of the cross-sectional subject Q12.

## Discussion

In this study, we wished to assess, whether medical students are *objectively* equipped to advices patients on phytotherapy. However, before addressing potential gaps in the knowledge on phytotherapy and thus enabling future physicians to advice patients, we first have to assess our students’ knowledge on official indications.

We used an open-worded request to write down the indication students associated with plants they used themselves in daily life. A drop-down menu might have prompted students to choose the correct “official indication” of the BfArM and might have thus resulted in higher congruency rates between the students’ answers and the official indications. However, the open approach offers us an insight into the “internalized” indications of our students. We used the indications offered by the participants and compared them per plant with the official indications of the BfArM. On the first glance, the previous self-assessment of physicians feeling not sufficiently equipped to advise patients (Ventola 2010) seems to be supported by our data: only a minority of students named the correct indication as stated by the BfArM. The highest congruency rates were found for single drug use of *Matricaria recutita* L., *Foeniculum vulgare* Mill., *Salvia officinalis* L. and *Mentha* x *piperita* L. Overall knowledge on indications of herbal mixtures was even less prominent. Here, the highest rates of correct indications were stated for *Foeniculum vulgare* Mill., *Matricaria recutita* L., *Pimpinella anisum* L. and *Plantago ovata* Forssk. Out of these plants, two—*Matricaria recutita* L. and *Mentha* x *piperita* L.—have in common that their level of evidence is discussed, respectively, in the national guideline for treating oral and abdominal discomfort (Layer et al. 2011; Leitlinienprogramm Onkologie der Arbeitsgemeinschaft der Wissenschaftlichen et al. 2020).

If we would translate the knowledge on the 25 plants surveyed into actual grades, students would receive for all except *Matricaria recutita* L. (knowledge of indication > 60%) a failing one. Therefore, we also assessed whether students are usually aware which plant is used for a certain symptom complex. Here, the overall rates of knowledge are higher. This shows that a basic understanding of the indications and applications of herbal medicine is present in our cohort. Still, we have to keep in mind that only *Matricaria recutita* L. and *Salvia officinalis* L. reached knowledge rates > 60%. Overall, students seem to lack deeper knowledge on official indications.

Taken together, our data fit previously published studies on medical students’ knowledge on phytotherapy and/or CAM. A similar observation was made by Yeo et al.: while medical students claimed to be familiar with certain CAM approaches (e.g., acupuncture), when tested, their knowledge on the CAM approaches was poor (Yeo et al. 2005). Another study among Turkish medical students made a similar observation concerning CAM approaches: only a minority of first-year (16%) and the fifth-year medical students (9%) were familiar with "The Regulation on Traditional and Complementary Medical Practices" published by the Ministry of Health of Turkey in 2014. The authors concluded that the awareness of CAM methods should be increased by further integrating CAM into medical curricula (Demir-Dora et al. 2020).

From our study and literature, we conclude that a deeper understanding of phytotherapy as a part of the CAM approaches is required. Therefore, where and how to start? To answer this question, we focused on whether phytotherapy as a part of naturopathy is taught at German medical schools. Approximately, the half of all students surveyed indicated that phytotherapy was either taught as a part of their faculties’ main curriculum or as part of electives offered. This is in line with our own, independent assessment of the curricula of the 38 medical schools in Germany. Overall, two-thirds of German medical schools indicated on their official websites that the cross-sectional subject Q12 (rehabilitation, physical medicine and naturopathy) is covered in their curricula. This information still does not convey, whether the focus of these faculties is equally divided between rehabilitation, physical medicine or/and naturopathy. The obvious knowledge gap we observed in the participating students argues for implementing a nation-wide a structured curriculum on phytotherapy. From other studies, we may even assume that students are interested in deepening their knowledge on CAM. A Swiss survey among medical students indicated that more than 70% of the students participating were favorable to establishing CAM education in Swiss medical schools. Students, CAM experts and conventional medical experts were mainly interested in acupuncture, homeopathy and phytotherapy (Nicolao et al. 2010). The need for a structured curriculum is further underlined by two studies: focusing on phytotherapy we know from a cross-sectional study in Nigeria, that medical students recognize misconceptions and misinformation on herbal remedies (Enwere 2009). Xu et al. described the high probability that medical students and later residents will encounter patients using herbals. However, their lack of knowledge and personal experience decrease their capability to rightfully advise patients concerning risks and benefits of herbal remedies (Xu and Levine 2008).

Taken together, we here present a glimpse into knowledge of medical students on herbals used for treating oral and abdominal discomfort. We observe a gap concerning the knowledge on official indications. Further integrating phytotherapy into the curricula of medical schools in Germany would be the next logical step to ensure a good foundation of knowledge enabling future physicians to correctly advice patients on herbal medicine.

In our study, we tried to assess knowledge of German medical students on phytotherapy. Due to study design, we were not able to correlate indications given by students with their level of medical education (both students of pre-clinical and clinical courses were able to participate). Hence, we are not able to address the question, whether students closer to graduation have a higher knowledge on phytotherapy. Furthermore, we worked under the premise that only if students stated the same indication as recommended by the BfArM for a certain plant, they knew how to use said herb. This approach led to very low congruency rates between students’ and the BfArM’s indications, while comparing symptom complexes of students’ and the BfArM’s indications shows higher congruency rates. Our chosen approach may at least partly ignore other possible, traditional indications of German folk medicine. We should also consider that students were asked to give indications they associated with the plants presented and not to write down “indications provided by the BfArM”. This led to a larger fundus of answers. However, a more precise, less open-worded assignment could perhaps lead to students concretizing their indications, thus leading to a higher rate of congruency between students’ and the BfArMs indications.

Taken together, our data fit previously published studies on medical students’ knowledge on phytotherapy and/or CAM. While we observed a small base of knowledge concerning the symptom complexes “oral discomfort” and “abdominal discomfort”, students are usually not aware of the officially provided indications. While German medical schools do integrate naturopathy into their curriculum, more in depth lessons seem to be required to ensure that future medical doctors are able to comprehensively advise their patients.

## Data Availability

All data concerning the study were included into the manuscript.
